# Effect of LED Irradiation with Different Red-to-Blue Light Ratios on Growth and Functional Compound Accumulations in Spinach (*Spinacia oleracea* L.) Accessions and Wild Relatives

**DOI:** 10.3390/plants14050700

**Published:** 2025-02-24

**Authors:** Tri Manh Le, Yuki Sago, Yasuomi Ibaraki, Kazuhiro Harada, Kenta Arai, Yuichi Ishizaki, Hitoshi Aoki, Mostafa Abdelrahman, Chris Kik, Rob van Treuren, Theo van Hintum, Masayoshi Shigyo

**Affiliations:** 1 Graduate School of Sciences and Technology for Innovation, Yamaguchi University, Yamaguchi 753-8511, Japan; e005wfv@yamaguchi-u.ac.jp (T.M.L.);; 2 Research and Development Division, Nichirei Foods Inc., Chiba 261-0002, Japan; 3 Institute of Genomics for Crop Abiotic Stress Tolerance, Department Plant and Soil Science, Texas Tech University, Lubbock, TX 79409, USA; mosabdel@ttu.edu; 4 Centre for Genetic Resources, The Netherlands, Wageningen University and Research, 6708 PB Wageningen, The Netherlandstheo.vanhintum@wur.nl (T.v.H.)

**Keywords:** plant factory, plant growth, chlorophyll, carotenoid, sugar content, amino acid content

## Abstract

The utilization of red and blue light-emitting diode (LED) lights for cultivating leafy vegetables in closed plant factories has increased in recent years. This study examined the growth and biosynthesis of functional compounds in twelve *Spinacia* accessions, including cultivars and wild relatives, under the irradiation of fluorescent light and three different red-to-blue LED light combinations (red:blue = 1:1, 1:3, and 3:1). Results showed that, except for the three examined Japanese cultivars, the fresh weight of most spinach accessions increased when red light comprised 50–75% of the light’s spectral composition. This indicated the vital role of the red-light photoreceptor phytochrome in inducing plant growth. The contribution of blue-light photoreceptors was also notable. Significant variations in the accumulation of amino acids and sugars were observed in specific accessions. The effects of spectral photons on the primary metabolite pathways were probably the leading causes of these variations. Some critical enzymes in the Gamma-aminobutyric acid (GABA) shunt cycle and the asparagine and glycolysis pathways were suggested as rate-limiting enzymes, which determined the biosynthesis of functional compounds. Among the examined *Spinacia* accessions, ‘CGN09429’, ‘CGN09511’, and the wild *S. turkestanica* ‘CGN25013’ were identified as potential breeding materials, while red:blue = 1:1 was determined as the optimal red-to-blue ratio for spinach growth in a closed-cultivation system.

## 1. Introduction

The growth and development of plants are significantly influenced by red and blue light, which play critical roles in photosynthesis and photomorphogenesis [1]. Red light is particularly effective in promoting several light-dependent reactions in photosynthesis and plays a key role in processes such as stem elongation, leaf expansion, and flowering. Blue light, on the other hand, affects stomatal conductance, phototropism, and the relocation of chloroplasts under certain conditions. These two types of light are absorbed by chlorophyll and carotenoids, which have maximum absorption peaks at specific wavelengths. The emergence of light-emitting diodes (LEDs) as a promising light source for horticulture production, offering selectable and narrow-spectrum emission, high energy-usage efficiency, and a long consumption time, opens up new possibilities for plant cultivation and nutrition research.

Spinach *(Spinacia oleracea* L.) is widely regarded as an important food crop due to its rich nutritional composition, which includes vitamins and minerals, as well as phytochemicals and bioactive compounds that promote health benefits beyond essential nutrition [2,3]. Based on their geographical origin, *S. oleracea* can be divided into two main groups: the eastern group and the western group [4]. Eastern cultivars feature narrow, hastate leaves that are smooth and often have an appetizing taste, but they are prone to bolting. In contrast, western cultivars have rounder leaves that are enlarged and exhibit a savoy texture, with a delayed tendency to bolt [5]. Additionally, the genus *Spinacia* includes two wild species, *S. turkestanica* and *S. tetrandra*, that are morphologically close to *S. oleracea* [6]. These three species are considered to form the primary gene pool for spinach [7]. The wild species interbreed with cultivated spinach, providing crucial genetic resources for introducing novel traits to enhance spinach varieties, particularly for commercial cultivars intended for growth under artificial irradiation. Numerous studies have emphasized the impact of red and blue light on the growth of spinach. However, there are still divergent viewpoints. The shoot dry weight of spinach grown under sole blue light was recorded to be much lower than those grown under white and sole red light [8]. Some studies suggest that a decrease in the red-to-blue light ratio generally decreases plant biomass, leaf length, width, and number of leaves [9,10]. Additionally, the importance of additional blue light to enhance the growth of spinach has been emphasized [11]. Even though there are some reports about the effects of different spectral compositions on the carotenoids or phenolic compounds in spinach [11,12,13], other functional compounds, such as amino acids or sugar content, have not been exhaustively studied. As amino acids and sugars play vital roles in plant physiology and contribute to the taste of leafy vegetables, it becomes possible to control the growth and taste of spinach for commercial production by applying suitable light conditions. Given the limited number of studies conducted and the fact that the focus of these studies on cultivated spinach has neglected the possibility that important genetic resources can be found in the two crossable wild relatives of spinach [14,15], further research on the suitable red and blue ratio for spinach cultivation is crucial. Therefore, this study aims to (1) examine how different combinations of red (R) and blue (B) LEDs impact the growth, phytopigments, amino acid content, and sugar content of various spinach accessions and wild relatives in a closed production system; and (2) determine the optimal red-to-blue ratio for each accession to guide further studies.

## 2. Results

### 2.1. Growth Features

**Figure 1:**
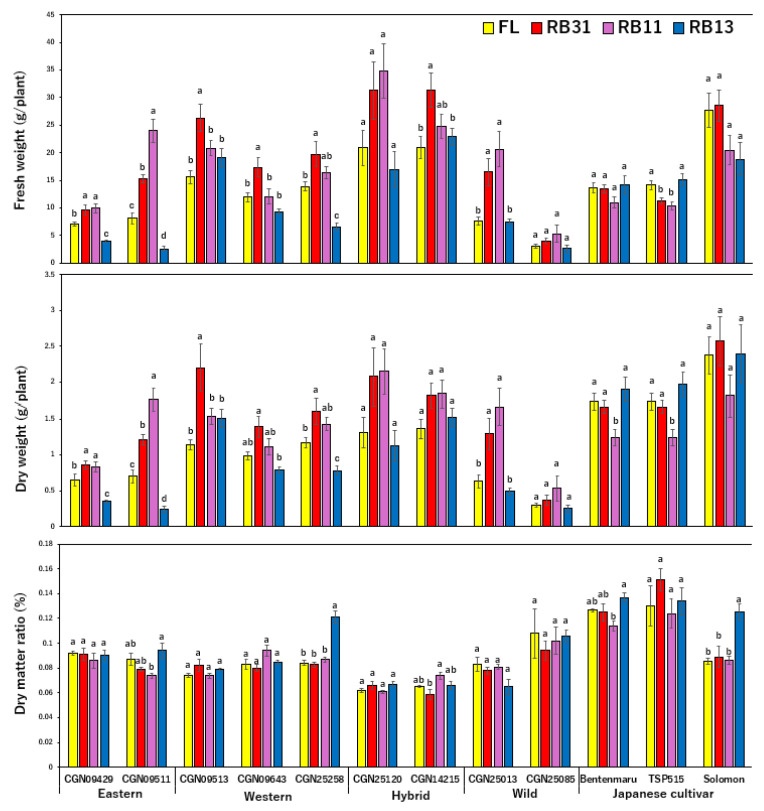
Effects of different light conditions on the growth features of spinach accessions and wild relatives (n = 6–10). Error bars represent the standard error (±SE). A one-way ANOVA was performed to determine significant differences in the effect of light on each accession. Different letters denote significant differences within one single accession by the post-hoc SNK test (*p* < 0.05).

**Figure 2:**
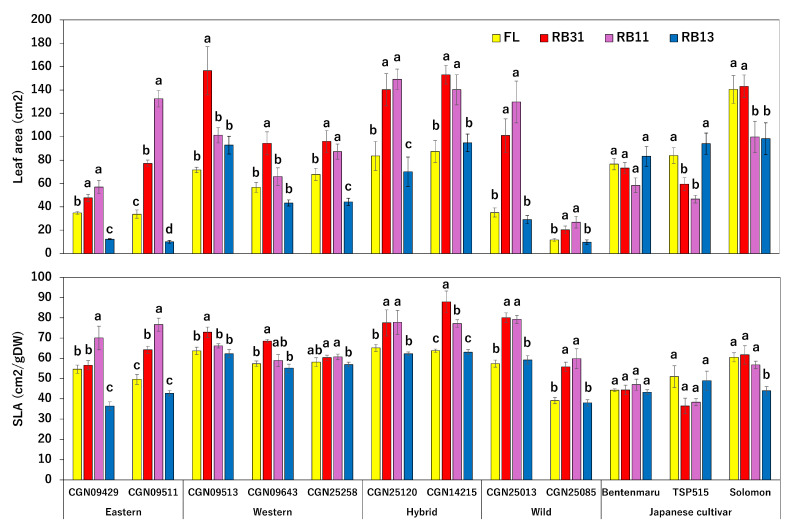
Effects of different light conditions on the leaf area and the specific leaf area (SLA) of spinach accessions and wild relatives (n = 3). Error bars represent the standard error (±SE). A one-way ANOVA was performed to determine significant differences in the effect of light on each accession. Different letters denote significant differences within one single accession by the post-hoc SNK test (*p* < 0.05).

The Japanese spinach cultivar ‘Solomon’ and ‘CGN25120’ exhibited the highest fresh weights (Figure S1). In contrast, the wild accession *S. tetrandra* ‘CGN25085’ showed the lowest yield. Among the CGN accessions, light conditions considerably influenced the fresh weights of spinach in both eastern and western groups (Figure 1). In general, CGN spinach achieved higher yields under RB11 and RB31 conditions. Specifically, the fresh weights of ‘CGN09513’ and ‘CGN09643’ were highest under RB31, while ‘CGN09511’ achieved its maximum fresh weight under RB11 (Figure 1). Other CGN accessions did not show significant differences between the two ratios. Meanwhile, a decline in fresh weight under strong blue light in RB13 was observed in eight of nine CGN accessions. The fresh weight of ‘CGN09429’, ‘CGN09511’, and ‘CGN25258’ under the RB13 were lower than those under the control condition. Interestingly, ‘TSP515’ was the only Japanese cultivar affected by changes in the red:blue ratio, with its fresh weight decreasing under both RB31 and RB11 conditions. Although trends in dry weight followed those of fresh weight (Figure 1), the dry matter ratios of certain accessions, such as ‘CGN25258’ and ‘Solomon’, significantly increased with greater blue light intensity. Concerning the leaf area indexes, leaf area was increased under the irradiation of RB31 or RB11 in all nine CGN accessions and ‘Solomon’ (Figure 2). ‘TSP515’ exhibited he largest leaf area under RB13, while there was no significant change in ‘Bentenmaru’. The specific leaf area was lowest under the RB13 condition in ten accessions, including all CGN accessions and ‘Solomon’. The impact of light on the specific leaf area was not significant in ‘Bentenmaru’ and ‘TSP515’.

### 2.2. Phytopigment Content

**Figure 3:**
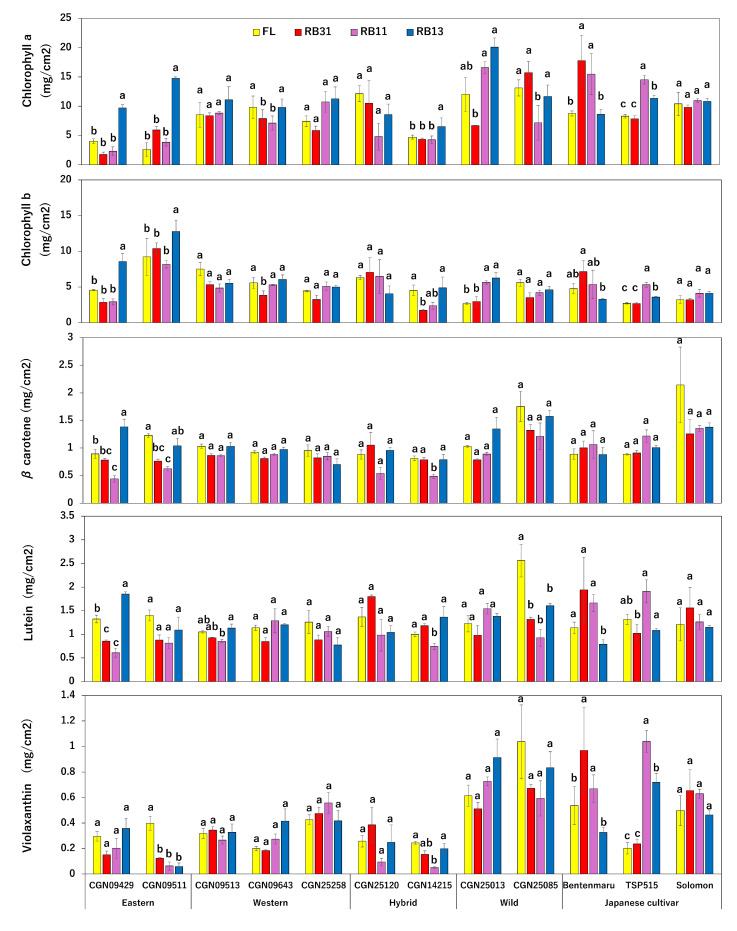
Effects of different light conditions on the phytopigment content of spinach accessions (n = 3). Error bars represent the standard error (±SE). A one-way ANOVA was performed to determine significant differences in the effect of light on each accession. Different letters denote significant differences within one single accession by the post-hoc SNK test (*p* < 0.05).

The concentrations of chlorophyll a and chlorophyll b were highest in the eastern accessions and ‘CGN14215’ under the RB13 condition (Figure 3 and Table S1). The two Japanese cultivars, ‘TSP515’ and ‘Bentenmaru’, recorded the highest values for both types of chlorophyll under RB11 and RB31, respectively. In contrast, the chlorophyll concentrations in ‘CGN09643’, ‘CGN25013’, and ‘CGN25085’ were the lowest under the RB31 irradiation condition. There was no significant variation in the chlorophyll content of ‘CGN09513’, ‘CGN25258’, ‘CGN25120’, and ‘Solomon’.

Regarding carotenoid concentrations, both genetic factors (accession) and environmental factors (light condition) had a significant impact on total carotenoid content per unit area (Figure 3). However, upon closer examination, only the concentration of *β*-carotene was affected by both factors and their interaction. Additionally, while genetic factors, along with their interaction with environmental factors, significantly influenced the concentrations of lutein and violaxanthin, the level of neoxanthin was affected solely by genetic factors. Notably, the RB13 condition enhanced the concentrations of *β*-carotene and lutein in the eastern accessions, including ‘CGN09429’ and ‘CGN09511’ (Figure 3 and Table S1). The RB11 condition significantly reduced the concentrations of *β*-carotene, lutein, and violaxanthin in ‘CGN14215’. In contrast, the RB11 condition notably increased the concentrations of lutein and violaxanthin in ‘TSP515’. Among the three red-to-blue ratios, both RB31 and RB11 significantly enhanced the concentration of lutein and violaxanthin per unit area, whereas RB13 had the opposite effect compared to the control condition. Significant variation was not recorded in other accessions.

### 2.3. Sugar Content

**Figure 4:**
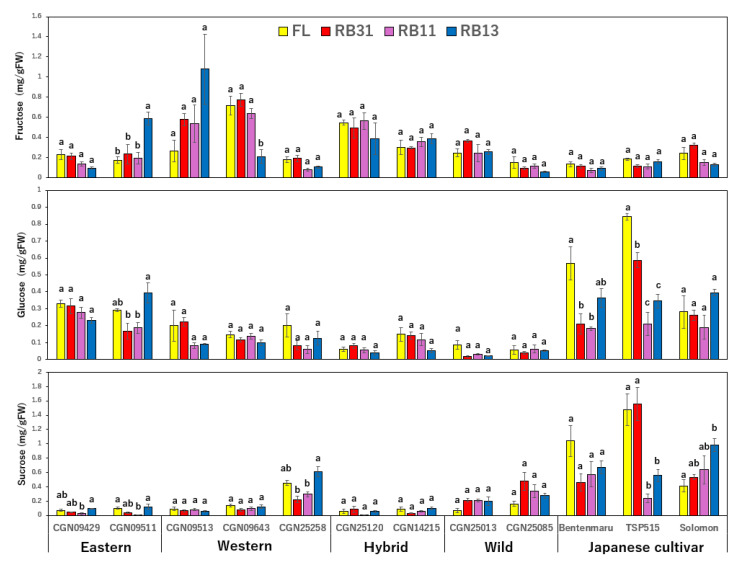
Effects of different light conditions on the sugar content of spinach accessions and wild relatives (n = 3). Error bars represent the standard error (±SE). The one-way ANOVA was performed to determine significant differences in the effect of light on each accession. Different letters denote significant differences within one single accession by the post-hoc SNK test (*p* < 0.05). FL: fluorescent irradiation; RB31: red/blue = 3/1; RB11: red/blue = 1/1; RB13: red/blue = 1/3.

Different groups with varying accessions exhibited diverse trends in sugar accumulation. Three Japanese cultivars displayed high levels of glucose and sucrose (Figure S3), whereas the wild accessions had a significantly lower sugar content than those of the other groups. The sugar concentrations in hybrid and wild accessions remained unchanged under the four examined treatments (Figure 4). In other accessions, RB31 and RB11 treatments tended to suppress glucose and sucrose content. Glucose levels were reduced in two Japanese cultivars, ‘Bentenmaru’ and ‘TSP515’, under RB11 irradiation. Additionally, RB11 decreased the sucrose concentration in ‘CGN09429’, ‘CGN09511’, ‘CGN25258’, and ‘TSP515’. Conversely, RB13 increased the concentration of all sugars in ‘CGN09511’ and sucrose levels in ‘CGN09429’ and ‘Solomon’.

### 2.4. Amino Acid Content

**Figure 5:**
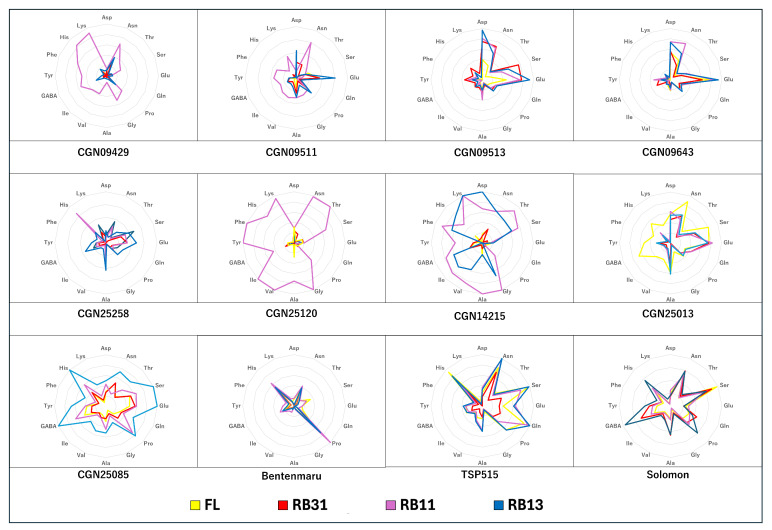
Changes in amino acid content in each spinach accession under different light conditions. FL: fluorescent irradiation; RB31: red/blue = 3/1; RB11: red/blue = 1/1; RB13: red/blue = 1/3. Asp: aspartic acid; Asn: asparagine; Thr: threonine; Ser: serine; Glu: glutamic acid; Gln: glutamine; Pro: proline; Gly: glycine; Ala: alanine; Val: valine; Ile: isoleucine; GABA: gama aminobutyric acid; Tyr: tyrosine; Phe: phenylalanine; His: histidine; Lys: lysine.

There were significant differences in the amino acid content among the twelve *Spinacia* accessions (Table S2). It was also indicated that changing red:blue ratios can modify the biosynthesis of amino acids in spinach. Remarkably, the impact of the interaction between accession and light condition was significant in all examined amino acids, leading to the varying responses of accessions to specific light condition. Light conditions did not significantly affect the amino acid content in Japanese cultivars and wild species. However, in spinach accessions from the eastern group, the amino acid content was greatly influenced, particularly by the RB11 ratio. Irradiation with the RB11 ratio resulted in a notable increase in several amino acids, including glycine, isoleucine, lysine, phenylalanine, threonine, tyrosine, and valine. Additionally, the concentrations of histidine and proline increased in ‘CGN09429’, along with rises in alanine, asparagine, and GABA levels in ‘CGN09511’ (Figure 5). In contrast, aspartic acid and glutamine concentrations in ‘CGN09511’ were lowest under the RB11 ratio and highest under the RB13 ratio. The hybrid accessions showed trends similar to those in the eastern group, with RB11 enhancing the production of alanine, glycine, histidine, isoleucine, lysine, phenylalanine, threonine, and valine in ‘CGN14215’. In ‘CGN25120’, all amino acids increased in concentration under RB11 irradiation, except for aspartic acid, glutamic acid, and glutamine (Figure 5). In the western accession group, only GABA concentrations rose with the RB31 ratio in ‘CGN09513’ and ‘CGN09643’. On the other hand, ‘CGN25258’ showed the highest accumulation of several amino acids under irradiation with the RB13 ratio.

## 3. Discussion

### 3.1. Growth Features

The interaction between light conditions and each spinach accession reveals a complex and nuanced relationship, highlighting the significant role of genetic background in shaping responses to varying red:blue light ratios. Among the twelve accessions, cultivated varieties such as ‘Solomon’ and ‘CGN25120’ consistently exhibited higher fresh weights, demonstrating their superior adaptability to light conditions compared to the wild accession ‘CGN25085’, which showed the lowest yield across all treatments. Light conditions significantly influenced growth, with most CGN accessions achieving optimal fresh weights under RB11 and RB31, suggesting that balanced or red-dominated light spectra are generally favorable for spinach growth. For instance, ‘CGN09513’ and ‘CGN09643’ thrived under RB31, while ‘CGN09511’ performed best under RB11, indicating accession-specific optima for light conditions. These results align with the findings of prior studies [14,16], and can be explained by the widely recognized growth-promoting impact of red light [17]. Red light interacts with specific photoreceptors, known as phytochromes, which play a critical role in regulating plant development. Upon absorbing red photons, phytochrome molecules transition from their inactive Pr form to the active Pfr form, triggering the activation of genes responsible for cell expansion. This process promotes the development of new leaves, enhances light capture, and ultimately increases photosynthesis rates. Exceptions such as ‘TSP515’, a Japanese cultivar, showed reduced fresh weight under both RB31 and RB11, suggesting its unique sensitivity to changes in red:blue ratios.

On the other hand, strong blue light led to a decline in fresh weight in eight out of nine CGN accessions. Research has shown that while blue light has the potential to enhance photosynthetic efficiency per unit leaf area, it may also reduce overall radiation capture, thereby limiting plant growth and productivity when combined with red light, as observed in lettuce cultivation [18]. A reduction in radiation absorption and subsequent growth inhibition typically occur when blue light is combined with other wavelengths [19]. These different responses between plants grown under monochromatic blue light and those exposed to blue and red dichromatic light are attributed to complex phytochrome–cryptochrome interactions, which regulate gene expression, plant growth, and development [20]. Additionally, blue photons are partially absorbed by inactive (e.g., anthocyanin) and accessory (e.g., carotenoids) pigments that do not contribute to energy transfer to chlorophyll reaction centers, resulting in lower photosynthetic efficiency as compared to red photons [21]. Moreover, studies on single-leaf photosynthesis have demonstrated that blue photons are utilized less efficiently than red photons [22]. The consensus is that blue light primarily affects photosynthetic efficiency through photomorphogenesis, which was shown clearly in this study. Most accessions, including all CGN accessions and ‘Solomon’, showed decreased leaf area under RB13. The absorption of the blue light by the photoreceptors cryptochrome and phototropin usually causes a decrease in leaf area and stem elongation by inhibiting cell division and expansion [17,23,24]. The effect of blue light on leaf morphology was also reflected by the decrease in specific leaf area (SLA) value in ten *Spinacia* accessions under the RB13 condition, indicating thicker or denser leaves under high blue light.

These findings collectively demonstrate that the interaction between light conditions and accessions is highly accession-specific, with each genotype exhibiting distinct optima and sensitivities to light spectra. This underscores the importance of the relationship between light environments and the genetic background of each accession to maximize growth and productivity, particularly in controlled agricultural systems such as greenhouses or vertical farms.

### 3.2. Phytopigment Content

Chlorophylls and carotenoids are the two main phytopigments found in plants. They are essential for capturing light, which is crucial for the process of photosynthesis. Chlorophylls primarily absorb light from the red and blue regions of the spectrum, while carotenoids are particularly effective at absorbing blue and green light. Agarwal noted that total chlorophyll content, the chlorophyll a to chlorophyll b ratio, carotenoid content, and the chlorophyll to carotenoid ratio remained stable under red to blue (R:B) light ratios of 25:75, 50:50, and 75:25 [13]. When nitrogen was adequately supplied, various combinations of blue, red, and green light did not significantly affect the carotenoid and chlorophyll content in spinach [25]. However, in this study, we evaluated chlorophyll and carotenoid content in terms of concentration per unit area. The results indicated that phytopigments tended to accumulate at higher densities under a blue-dominant light source in certain accessions. This accumulation can be attributed to the lower specific leaf area (SLA) observed under the R:B ratio of 1:3, which resulted in thicker and more compact leaves, leading to a higher concentration of phytopigments on the leaf surface. This finding is supported by previous research on the impact of different doses of blue light on cucumber seedlings [26]. According to the ANOVA results, the contents of three carotenoids—lutein, neoxanthin, and violaxanthin—were not significantly affected by the light source. However, the levels of lutein and violaxanthin varied when considering the interaction between genetic factors and light sources, contributing to the overall variation in total carotenoid content.

The twelve *Spinacia* accessions exhibited different responses to varying red-to-blue light ratios. The eastern accessions, along with ‘CGN14215’, may have a genetic predisposition that allows them to optimize chlorophyll synthesis under high blue light conditions. In contrast, the Japanese cultivars (‘TSP515’ and ‘Bentenmaru’) displayed a preference for either balanced (RB11) or red-dominant (RB31) light, suggesting they are better adapted to environments with lower availability of blue light. Some accessions showed low chlorophyll content under RB31, which may indicate photo-inhibition or a reduction in chlorophyll synthesis when exposed to red-dominant light, a condition that is less efficient for producing chlorophyll b. Additionally, the reduction in carotenoid levels observed under RB11 in ‘CGN14215’ may reflect a stress response or a shift in pigment allocation in balanced light conditions. The lower carotenoid content noted under RB13 in some accessions could suggest a trade-off between light capture and photoprotection, since exposure to blue light can also lead to photo-inhibition if it is not properly balanced with red light.

### 3.3. Sugar Content

Cultivated accessions, particularly the three Japanese cultivars, exhibited higher levels of glucose and sucrose compared to wild accessions, underscoring their suitability for commercial use due to their enhanced sweetness. In contrast, wild accessions consistently showed lower sugar content, reflecting their genetic predisposition for reduced sweetness. The red:blue (R:B) ratios had a notable but accession-specific impact on sugar accumulation. While fructose content remained relatively stable across light treatments, the interaction between accession and light condition significantly influenced fructose, glucose, and sucrose levels, indicating the importance of genetic background in shaping responses to light spectra.

Among the cultivated accessions, RB31 and RB11 generally suppressed glucose and sucrose content. For example, glucose levels decreased in the Japanese cultivars ‘Bentenmaru’ and ‘TSP515’ under RB11, while sucrose concentrations declined in ‘CGN09429’, ‘CGN09511’, ‘CGN25258’, and ‘TSP515’ under the same treatment. This suggests that balanced or red-dominated light conditions may inhibit sugar accumulation in certain accessions, possibly due to altered metabolic pathways or resource allocation under these spectra. In contrast, RB13, which emphasizes blue light, increased sugar content in specific accessions. For instance, ‘CGN09511’ showed elevated levels of all sugars under RB13, while ‘CGN09429’ and ‘Solomon’ exhibited higher sucrose concentrations. This indicates that blue light may stimulate sugar accumulation in some accessions, potentially by enhancing the activity of enzymes involved in carbohydrate metabolism, such as fructose biphosphate (FBP) enzymes [27,28]. These enzymes play a critical role in gluconeogenesis and are known to be regulated by light [29], which could explain the observed variations in fructose and sucrose levels under different light conditions.

### 3.4. Amino Acid Content

The biosynthesis of amino acids was notably modified by changes in red:blue light ratios, with the interaction between accession and light condition playing a critical role in shaping these responses. While light conditions did not significantly affect amino acid content in Japanese cultivars and wild species, accessions from the eastern and western groups, as well as hybrid accessions, exhibited pronounced changes in amino acid accumulation under specific light treatments. These findings highlight the complex interplay between genetic background and environmental factors in regulating amino acid metabolism.

**Figure 6:**
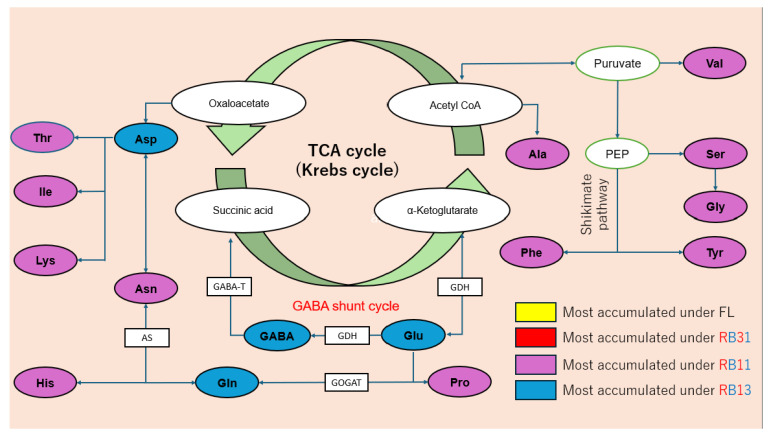
Relationship between the TCA cycle and the biosynthesis pathways of amino acids. Different colors indicate the light condition in which a specific amino acid was most accumulated. Squares represent relevant enzymes.

The RB11 ratio had a particularly strong impact, significantly increasing the concentrations of several amino acids, including glycine, isoleucine, lysine, phenylalanine, threonine, tyrosine, and valine (Figure 6). ‘CGN09429’ showed elevated levels of histidine and proline under RB11, while ‘CGN09511’ exhibited increased alanine, asparagine, and GABA concentrations. However, ‘CGN09511’ also displayed contrasting responses, with aspartic acid and glutamine levels being lowest under RB11 and highest under RB13, suggesting that intense blue light may enhance the biosynthesis of these specific amino acids in this accession. Hybrid accession ‘CGN25120’ showed increased concentrations of nearly all amino acids under RB11, except for aspartic acid, glutamic acid, and glutamine, which were unaffected. In contrast, the western group exhibited more limited responses, with only GABA concentrations increasing under the RB31 ratio in ‘CGN09513’ and ‘CGN09643’. Interestingly, ‘CGN25258’ accumulated the highest levels of several amino acids under RB13, indicating that this accession may have a unique metabolic adaptation to strong blue light.

The observed variations in amino acid content can be linked to the roles of key amino acids in metabolic pathways and flavor profiles. Aspartic acid, glutamic acid, GABA, and glutamine were identified as the most abundant amino acids in spinach, with glutamic acid and aspartic acid contributing to the savory flavor and playing critical roles in nitrogen metabolism. Glutamic acid, glutamine, and GABA are interconnected through the GABA shunt cycle, which is closely tied to the TCA cycle, a central pathway for energy production and growth [30,31]. Aspartic acid, on the other hand, serves as a key nitrogen source and is linked to the TCA cycle via its conversion to oxaloacetate [32]. The promotion of amino acid biosynthesis under RB11 and RB13 conditions suggests that these light ratios enhance the activity of metabolic pathways involved in amino acid production. The differential responses of accessions to light conditions can be attributed to variations in the activation of key enzymes, such as glutamate dehydrogenase (GDH) and glutamate decarboxylase (GAD), which are involved in the biosynthesis of glutamic acid and GABA. In accessions like ‘CGN09511’ and ‘CGN25258’, intense blue light (RB13) may suppress the activity of these enzymes, reducing the production of succinic acid and α-ketoglutarate and thereby decreasing TCA cycle efficiency and growth. In contrast, accessions like ‘CGN25120’ and ‘CGN14215’ may possess metabolic adaptations that allow them to maintain or enhance amino acid biosynthesis under RB11 and RB13 without compromising growth.

### 3.5. Overall Review

**Figure 7:**
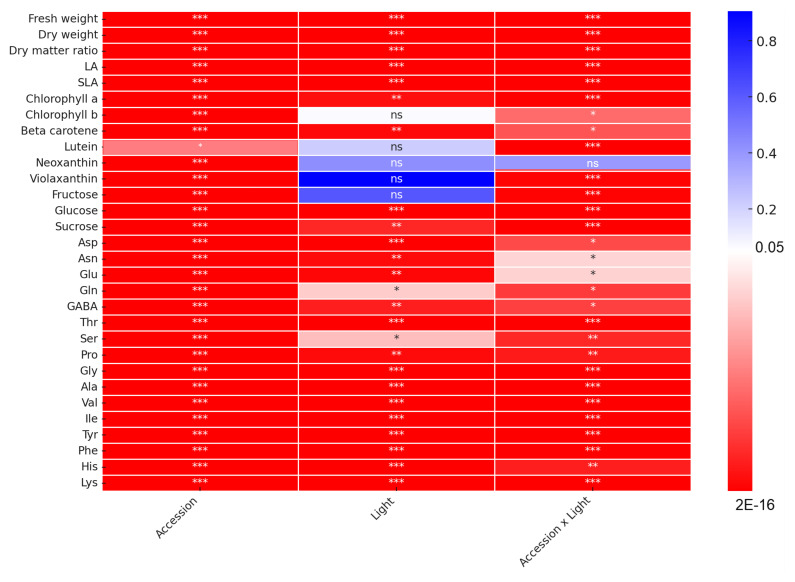
Results of the two-way ANOVA, demonstrating the *p*-values of genetic factor (Accession), environmental factor (Light), and their interaction. Red highlight indicates significance (*p* < 0.05). Asterisks denote significance level; ‘***’: *p* = 0–0.001; ‘**’: *p* = 0.0011–0.01; ‘*’: *p* = 0.011–0.05; ns: no significant difference.

**Figure 8:**
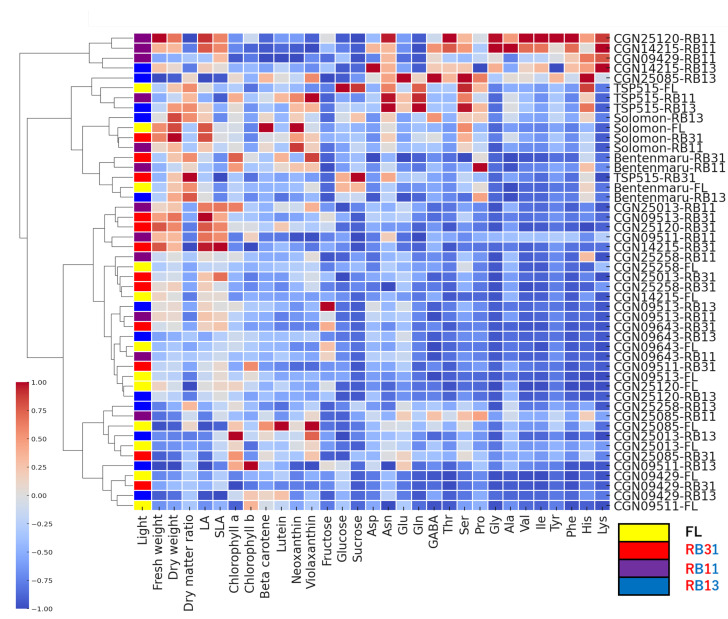
Overall assessment of *Spinacia* accessions across light conditions.

The results of this study showed that the interaction between accession and light can significantly affect the growth and the biosynthesis of functional compounds in spinach (Figure 7), and the response of spinach accessions to different red:blue light ratios varied significantly based on genetic background (Figure 8). The combinations of hybrid accessions with the RB11 condition, ‘CGN25120-RB11’ and ‘CGN14215-RB11’, resulted in the highest values for biomass and amino acid content across all combinations. A notable increase in biomass accumulation was also observed in ‘Solomon-RB31’, ‘Solomon-FL’, ‘Solomon-RB11’, ‘CGN09513-RB31’, ‘CGN25120-RB31’, ‘CGN09511-RB11’, and ‘CGN14215-RB31’. The highest chlorophyll content was recorded in ’CGN25085-FL’, while the highest carotenoid content was found in ‘CGN09511-RB13’. ‘TSP515-RB31’ had the highest sugar concentration and exhibited a remarkable level of biomass accumulation. In general, RB11 can be considered to be the optimal ratio for all tested CGN *Spinacia* accessions, which promoted the growth and induced the synthesis of functional compounds at a certain level. RB31 and RB13 increased some parameters in specific accessions, suggesting that those results can be used to control the productivity and flavor of spinach to meet the requirements of the producers.

Wild accession ‘CGN25085’ (*S. tetrandra*) showed minimal responsiveness, maintaining consistent biomass, sugar, and amino acid content across all light treatments, likely due to its adaptation to stable natural environments. In contrast, ‘CGN25013’ (*S. turkestanica*) increased fresh weight and elevated chlorophyll and carotenoid levels under the irradiation of RB31 and RB11, making it a valuable genetic resource for breeding.

## 4. Materials and Methods

### 4.1. Plant Materials

Nine spinach accessions from the Center for Genetic Resources, The Netherlands were chosen from the gene bank at Wageningen University and Research in The Netherlands (Table 1). In order to maximize the level of genetic diversity, accessions were selected based on their geographical origins. These included seven accessions of spinach (*Spinacia oleracea* L.), including ‘CGN09429’, ‘CGN09511’, ‘CGN09513’, ‘CGN09643’, ‘CGN25258’, ‘CGN14215’, and ‘CGN25120’, and two accessions of closely related wild species, ‘CGN25013’ (*Spinacia turkestanica*) and ‘CGN25085’ (*Spinacia tetrandra*). Additionally, three Japanese cultivars (Jpn cultivar), ‘Bentenmaru’and ‘TSP515’ from Takii & Co., Ltd., and ‘Solomon’ from The Sakata Seed Corporation, were included for comparison. The eastern and western groups were distinguished by their morphological differences (Figure S4). Accessions in the eastern group had narrow, hastate leaves, while those in the western group featured round leaves with a savoy texture. The accessions in the hybrid group are the offspring of hybrids between eastern and western spinach.

### 4.2. Growing Conditions

The experiment took place in a deep-flow hydroponic system at the plant factory on Yoshida Campus, Yamaguchi University. The hydroponic solution used the Otsuka SA formula with an electrical conductivity (EC) of 2.0 dS/m and a pH level of 5.5–6.5. CO_2_ was added to the cultivation chamber using an air pump at a consistent concentration of 1200 ppmv, while the room temperature and relative humidity were maintained at 20 °C and 60% (±10%), respectively, with an air conditioning system installed within the plant factory.

### 4.3. Irradiation Pattern

**Figure 9:**
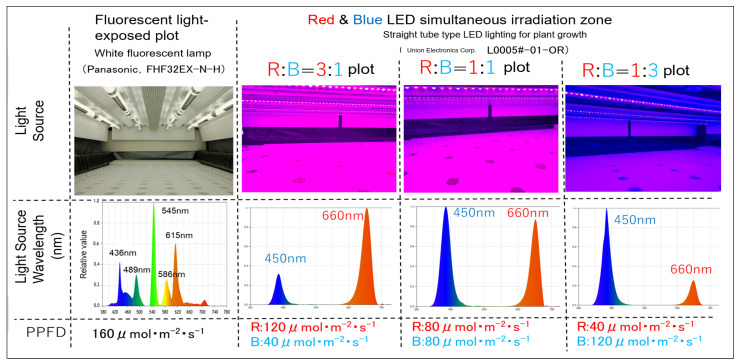
Spectral composition of the four irradiation treatments.

To estimate the effect of red and blue LED light on spinach, three red:blue ratios equaling 3:1, 1:1, and 1:3 (RB31, RB11, and RB13, respectively) were selected to irradiate simultaneously for 12 h (Figure 9). The light sources were red and blue LED lights from Union Electronics (#-01-OR). Red light peaked at 650 nm, whereas the peak wavelength of blue light was 450 nm. Fluorescent light (FHF32EX-N-H, Panasonic) with peaks ranging from 436 nm to 615 nm was used as a controlled treatment (FL). PPFD was set at 160 µmol/m^2^/s in all plots. To ensure uniform germination due to the high dormancy of *S. turkestanica* and *S. tetrandra*, before sowing, all seed coats were carefully removed using pliers. The seeds were then placed in wet towels inside trays, which were covered with plastic wrap that had a small gap for seed respiration. The trays were kept in a dark area within the cultivation room of the plant factory. Five days after sowing, germinated seeds were temporarily planted in urethane foam for three days under fluorescent illumination. Spinach seedlings were transplanted into hydroponic beds and exposed to four different light conditions with a 12 h day length eight days after planting. The plants were harvested on the 32nd day after sowing (32DAS) (Figure S5).

### 4.4. Plant Growth Measurement

Six to ten plants of the same size were selected from each irradiation plot on 32DAS. Their fresh weight (FW) was determined immediately after harvesting. Then, all samples were freeze-dried using a VD-250R Freeze Dryer for 72 h to collect dry matter and measure the dry weight (DW). The samples were ground into dry powder and used for subsequent experiments. ImageJ 1.54g and Agisoft Metashape 2.2.0 were utilized to measure leaf area (LA) from 3D phenotyping figures and images, then the specific leaf area (SLA) was determined as the ratio of leaf area to dry weight.

### 4.5. Phytopigment Content Measurement

A total of 20 mg of powder was measured into a 15 mL tube, and 2.5 mL of 100% acetone was added. The sample was then vortexed for 2 min, homogenized for 20 min, and centrifuged at 5000 rpm for 5 min. The supernatant was collected in a 5 mL light-shielded glass sample tube and filtered through a 0.20 µm membrane filter (Toyo Seisakusho Kaisha, Ltd., Chiba, Japan). Samples were stored in a freezer at −25 °C and used for analysis within three days. Identification of chlorophyll a, chlorophyll b, lutein, *β*-carotene, violaxanthin, and neoxanthin was based on HPLC retention time using a LiChroCART 250-4.0 LiChrospher 100 RP-18 (5 µm) column and a UV-VIS Detector L-7420 (Hitachi High-Tech Corporation, Ibaraki, Japan). The injected volume for each sample was 50 µL. The column temperature was maintained at 30 °C, and the flow rate was 1.0 mL/min. For the first 20 min, the mobile phase was 80% methanol, and for the next 30 min, a mixed solution of 80% methanol:ethanol = 1:1 was passed through the column. The detection wavelength was set at 435 nm.

### 4.6. Sugar Content Measurement

Ten mg of the sample was measured and placed in a 15 mL tube. Then, 2 mL of 70% ethanol was added. The tube was vortexed for 5 min, heated in a block constant temperature chamber set at 75 °C for 15 min, and sonicated for 5 min. The supernatant liquid was collected with a Pasteur pipette, filtered through a 0.45 µm membrane filter (Toyo Seisakusho Kaisha, Ltd., Chiba, Japan), collected in a vial, and used as the sample solution for HPLC analysis. The sample solution was stored at −25 °C. The HPLC analysis utilized the LiChrospher 100 NH2 250-4.0 column (KANTO CHEMICAL Co., Inc., Saitama, Japan) and the RID-10A detector (Shimadzu Corporation, Kyoto, Japan). The injected volume for each sample was 50 µL. The column temperature was maintained at 35 °C, and the flow rate was 0.8 mL/min. The mobile phase was 80% acetonitrile.

### 4.7. Amino Acid Content Measurement

Five mg of each sample was measured in 2 mL tubes, and then 1 mL of pH 2.2 lithium citrate (FUJIFILM Wako Pure Chemical Corporation, Hyogo, Japan) was added as the buffer solution. Samples were vortexed for 5 min and sonicated for 10 min. The supernatant was collected with a Pasteur pipette, filtered through a 0.20 µm membrane filter (Toyo Seisakusho Kaisha, Ltd., Chiba, Japan), and collected in a vial as the sample solution. The instrument used was the Shimadzu high-performance liquid chromatogram amino acid analysis system, with the RF-20A Spectro fluorescence detector (Shimadzu Corporation, Kyoto, Japan) used as the detector and the Li-type post-column derivatization method used as the internal standard method. Calibration curves were prepared for each measurement based on standard samples. The sample solutions were stored at −4 °C. Thirty-nine amino acids were analyzed, and those with a significant amount were selected for statistical analysis, including aspartic acid (Asp), asparagine (Asn), glutamic acid (Glu), glutamine (Gln), gamma-aminobutyric acid (GABA), serine (Ser), histidine (His), proline (Pro), tyrosine (Tyr), alanine (Ala), phenylalanine (Phe), glycine (Gly), lysine (Lys), isoleucine (Ile), valine (Val), and threonine (Thr).

The HPLC conditions were set as follows:

- **Column:** Shim-pack Amino-Li
- **Mobile phase:** Amino acid analysis mobile phase kit LI type
- **Mobile phase A:** Lithium ion, citric acid, 2-methoxyethanol, perchloric acid
- **Mobile phase B:** Lithium ion, citric acid, boric acid, hydroxide ion
- **Mobile phase C:** Lithium ion, hydroxide ion
- **Reaction solution A:** Sodium carbonate, boric acid, potassium sulfate, sodium hypochlorite
- **Reaction solution B:** Sodium carbonate, boric acid, potassium sulfate, OPA, ethanol, N-acetyl-L-cysteine
- **Flow rate (initial conditions):** 0.6 mL/min
- **Column temperature (initial conditions):** 39 °C
- **Detector:** Spectro fluorescence detector RF-20Axs
- **Detection wavelength:** 450 nm
- **Gradient:** Shimadzu high-performance liquid chromatography prominence amino acid analysis system

### 4.8. Statistical Analysis

The items measured and analyzed were subjected to analysis of variance using the statistical analysis software R 4.4.2. The significance of the impact of genetic factors (twelve *Spinacia* accessions) and the environmental factor (irradiation conditions), as well as their interaction, was tested by two-way ANOVA. Results (*p*-values) of the two-way ANOVA were visualized with a heat map to clarify the significant impact of each factor on parameters. Then, a one-way ANOVA was performed for each single accession, followed by the Student-Newman-Keuls (SNK) post-hoc test to determine which light condition made a significant difference in the examined parameters. For overall assessment of all accession–light condition combinations, a heat map was drawn with the normalized average values (ranging from −1.00 to 1.00). The heat maps were released with Python 3.13.2 (programming language).

## 5. Conclusions

This study highlights the significant influence of red-to-blue LED light ratios on the growth performance and functional compound accumulation in spinach and its wild relatives under controlled environments. Fresh weight was maximized when red light comprised 50–70% of the spectra, emphasizing the critical role of red light in enhancing biomass production through phytochrome-mediated pathways. In contrast, a high proportion of blue light generally suppressed growth, likely due to its effects on photomorphogenesis regulation. The study revealed notable variations in phytopigment content, with specific accessions responding differently to light treatments. There was no significant impact on the growth of the wild spinach ‘CGN25085’ (*S. tetrandra*) or Japanese cultivars ‘Bentenmaru’ and ‘Solomon’, and a growth-suppression effect of RB31 and RB11 was observed in ‘TSP515’. The response to light conditions varied among different accessions for various functional compounds. Notably, ‘CGN09429’, ‘CGN09511’, and wild spinach ‘CGN25013’ (*S. turkestanica*) were most influenced by different red/blue ratios. It was suggested that the variations in functional compound biosynthesis could be attributed to the impact of spectral photons on the activation of key enzymes in metabolic pathways. These findings are expected to enhance our knowledge of how light affects spinach. This could help determine the optimal red and blue light ratios for each spinach accession, ultimately aiding in developing specialized spinach varieties for plant factory cultivation.

## Figures and Tables

**Table 1:** List of twelve spinach accessions and their population group.

Accession	Population Group ^x^	Scientific Name	Origin ^y^
CGN09429	Eastern group	*Spinacia oleracea*	The Netherlands
CGN09511	Eastern group	*Spinacia oleracea*	Ethiopia
CGN09513	Western group	*Spinacia oleracea*	France
CGN09643	Western group	*Spinacia oleracea*	Iran
CGN25258	Western group	*Spinacia oleracea*	Ukraine
CGN25120	Hybrid group	*Spinacia oleracea*	Azerbaijan
CGN14215	Hybrid group	*Spinacia oleracea*	China
CGN25013	Wild group	*Spinacia turkestanica*	Uzbekistan
CGN25085	Wild group	*Spinacia tetrandra*	Georgia
Bentenmaru	Japanese cultivar	*Spinacia oleracea*	Japan
TSP515	Japanese cultivar	*Spinacia oleracea*	Japan
Solomon	Japanese cultivar	*Spinacia oleracea*	Japan

^x^ Categorized by the Center for Genetic Resources, Wageningen University & Research. ^y^ The country where the sample was collected or derived.

## Data Availability

Data are contained within the article and Supplementary Materials.

## Supplementary Materials

The following supporting information can be downloaded at: https://www.mdpi.com/article/10.3390/plants14050700/s1, Figure S1: Growth features of 12 spinach accessions; Figure S2: Phytopigment content of 12 spinach accessions; Figure S3: Sugar content of 12 spinach accession; Figure S4: Morphological difference of twelve spinach accessions; Figure S5: Growing schedule; Table S1: Phytopigment content; Table S2: Amino acid content.
