# Supplementary figures and images for: Effect of LED Irradiation with Different Red-to-Blue Light Ratios on Growth and Functional Compound Accumulations in Spinach (Spinacia oleracea L.) Accessions and Wild Relatives
Source: Plants (Basel). 2025 Feb 24;14(5):700. doi: 10.3390/plants14050700 (PMC11902216; doi:10.3390/plants14050700)

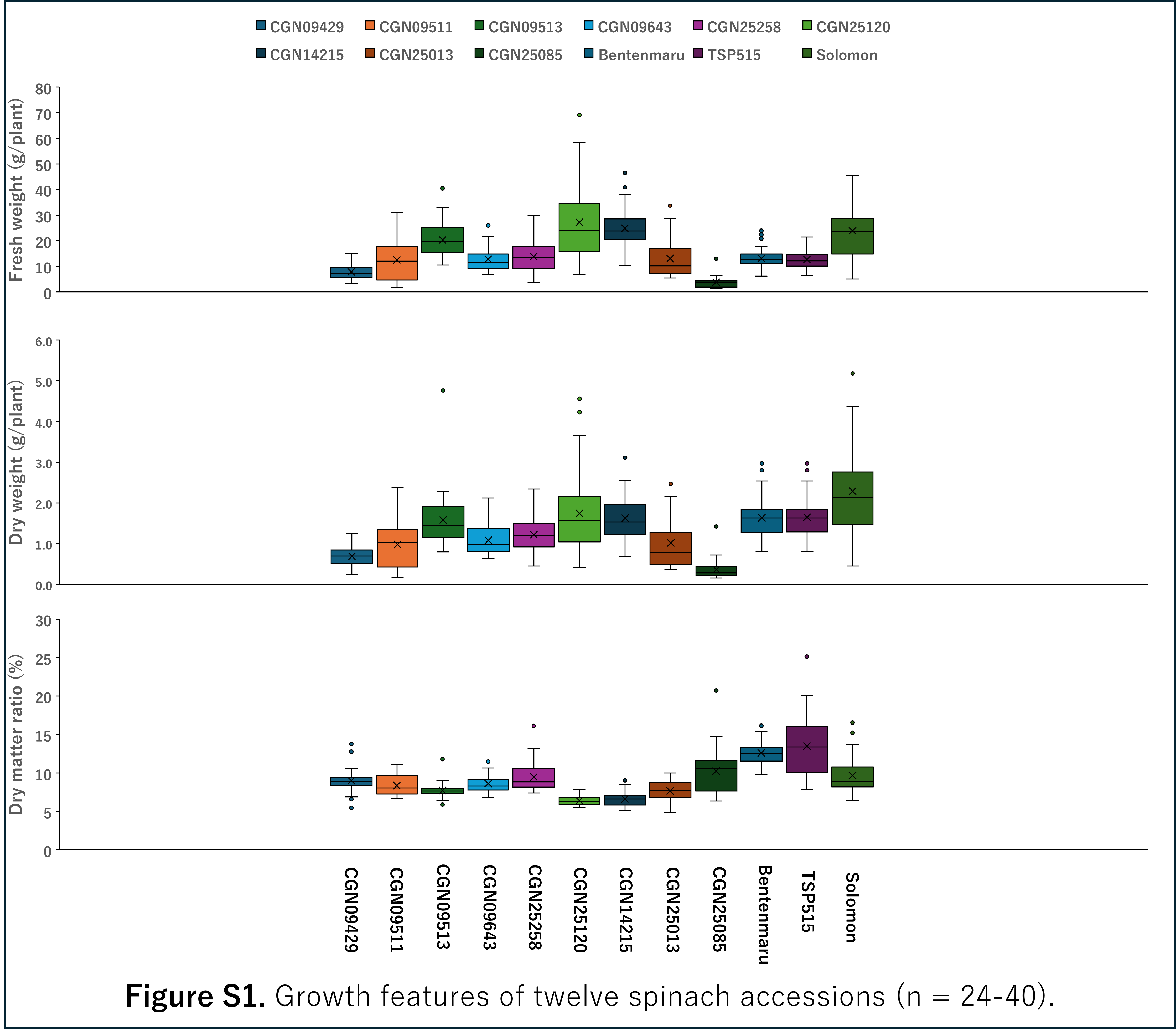

Supplement: Supplementary file 1 [file plants-14-00700-s001.zip › Figure S1. Growth features of 12 spinach accessions.png]

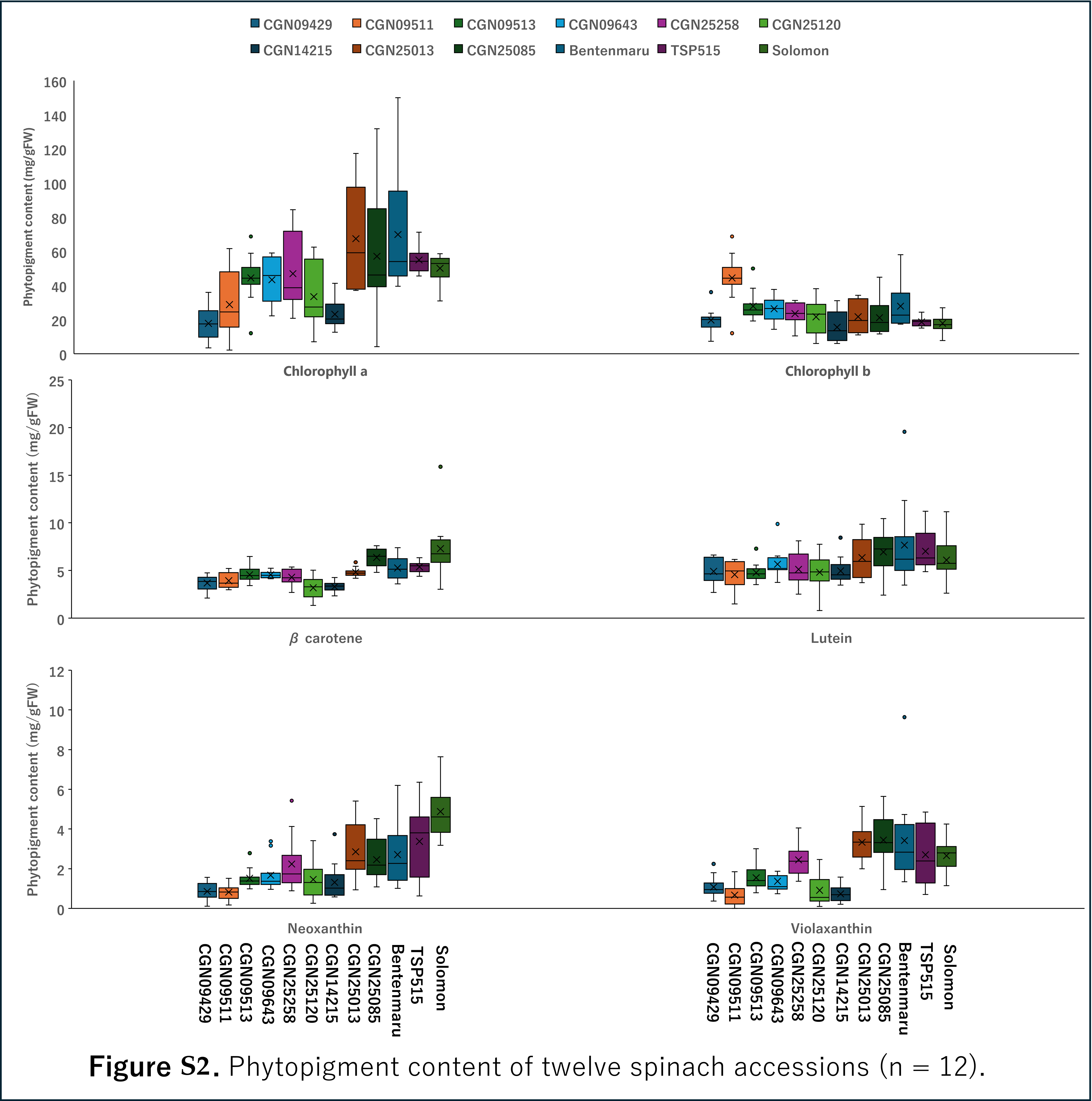

Supplement: Supplementary file 1 [file plants-14-00700-s001.zip › Figure S2. Phytopigment content of 12 spinach accessions.png]

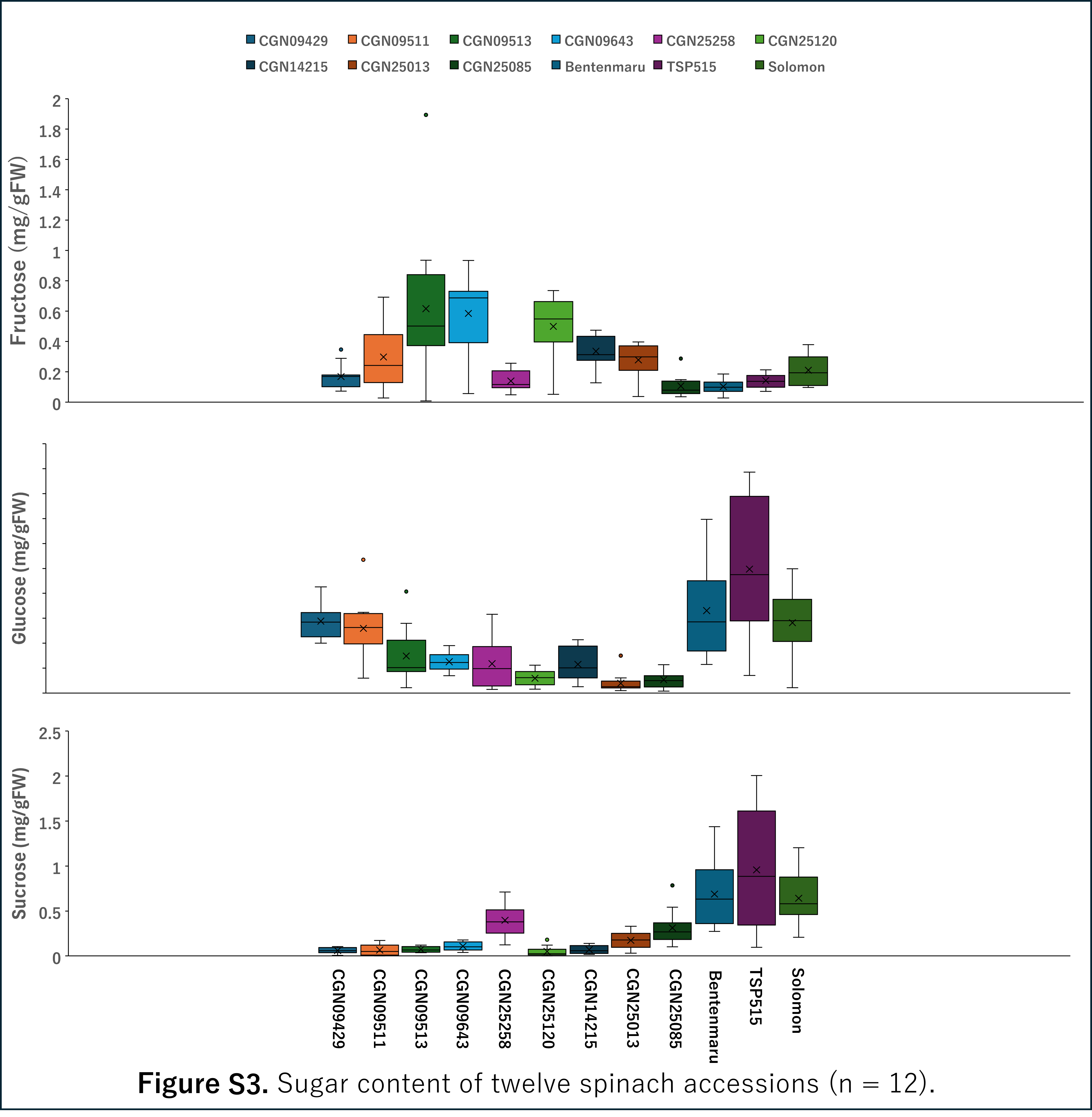

Supplement: Supplementary file 1 [file plants-14-00700-s001.zip › Figure S3. Sugar content of 12 spinach accessions.png]

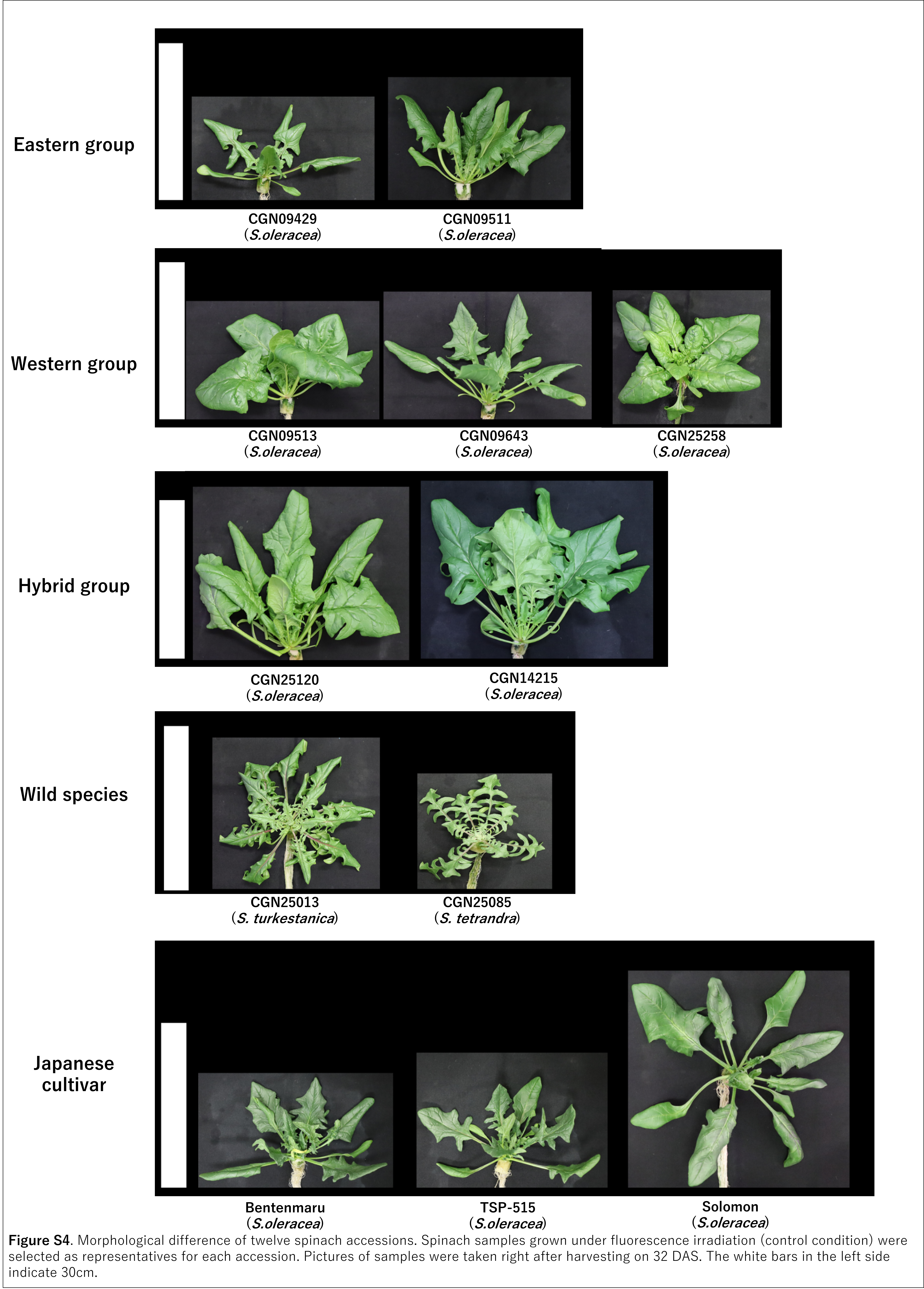

Supplement: Supplementary file 1 [file plants-14-00700-s001.zip › Figure S4. Morphological difference of twelve spinach accessions.png]

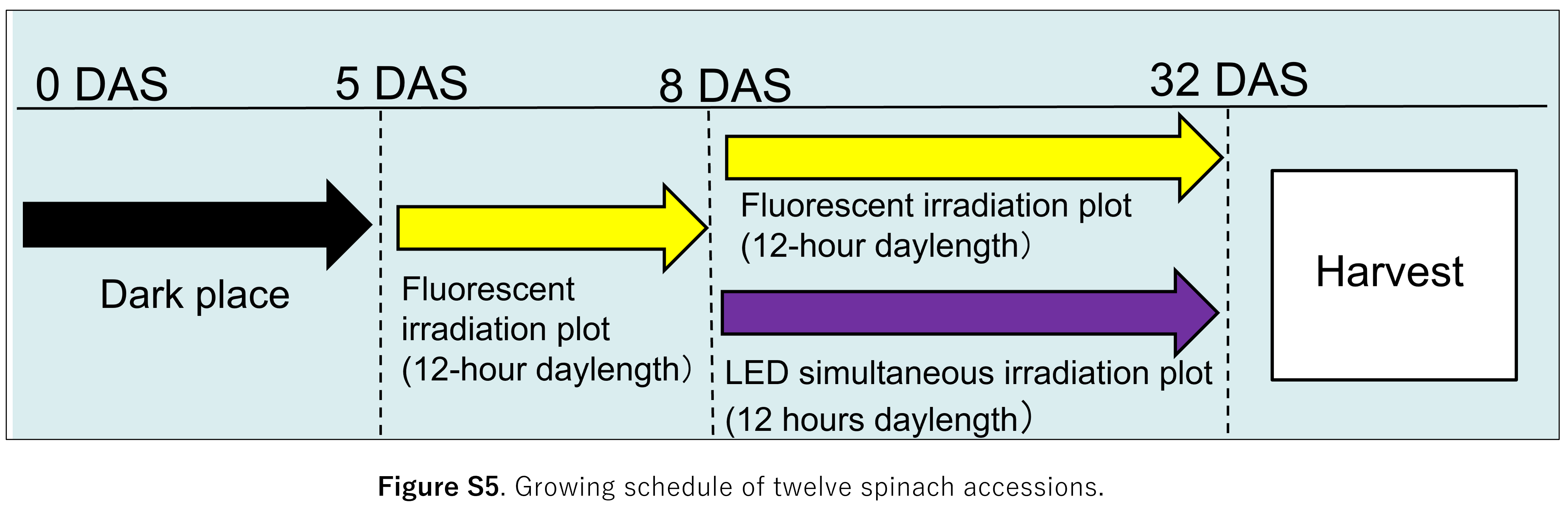

Supplement: Supplementary file 1 [file plants-14-00700-s001.zip › Figure S5. Growing schedule.png]
